# Association between student-teacher ratio and teachers’ working hours and workload stress: evidence from a nationwide survey in Japan

**DOI:** 10.1186/s12889-021-11677-w

**Published:** 2021-09-07

**Authors:** Masakazu Hojo

**Affiliations:** Department of Economics, Komazawa University, Tokyo, Japan

**Keywords:** Student-teacher ratio, Workload stress, Work hours, School teachers

## Abstract

**Background:**

Too long work hours of Japanese school teachers, along with an increasing number of teachers taking leave due to mental illness, are well known and recognized as a serious social problem. In order to prevent the spread of COVID-19 by reducing the density in the classroom, the Japanese government has decided to reduce the upper-limit of class size in primary schools after 2021, which is expected to result in lowering the student-teacher ratio. The aim of this study was to examine the association between student-teacher ratio, teacher work hours and teacher stress.

**Methods:**

Data on student-teacher ratio, teacher work hours, and teacher stress were obtained from a large-scale international survey conducted by OECD. The number of teachers participated in the survey was 3308 (primary school) and 3555 (lower-secondary school). After excluding teachers with missing observations, the analytic sample consisted of 2761 (primary school) and 3006 (lower-secondary school) teachers. Multivariate regression analysis was performed.

**Results:**

Regression results revealed that student-teacher ratio was positively correlated with total work hours and workload stress of teachers. In particular, teachers working in schools with high student-teacher ratio spent more time on time-consuming tasks such as marking/correcting student work and communication with parents or guardians. The coefficient estimates suggested that, on average, lowering the student-teacher ratio by five in lower-secondary school was associated with 2.8 hours shorter working hours per week (*p*<0.001).

**Conclusion:**

Our empirical results suggested that the class-size reduction policy starting in 2021 could reduce teacher stress and long work hours through the consequent decrease in student-teacher ratios.

## Background

It is well known that school teachers in Japan work too long hours. According to the results of the OECD Teaching and Learning International Survey, an international, large-scale survey of teachers conducted in 2018 (hereafter TALIS 2018), the working hours of school teachers in Japan were the longest among the participating countries. The average weekly working hours of junior high school teachers in Japan was 56.0 hours, which was not only much higher than the average of the participating countries, 38.3 hours, but was also the only value exceeding 50 hours among them [1].

The problem of stress and resulting burnout among teachers has been reported in different countries and is considered to be an international phenomenon [2–7]. In Japan, the percentage of school teachers taking leave due to mental illness among all teachers has increased more than fivefold from 0.11% in 1992 to 0.59% in 2018 [8, 9]. Existing studies found significant association between occupational stress and mental health [10, 11], long working hours and psychological distress [12], poor mental health and lower job satisfaction [13], and prolonged fatigue and both quantitative and qualitative workload [14], among school teachers in Japan. A recent large-scale survey conducted by the Ministry of Education, Culture, Sports, Science and Technology (MEXT) found significant correlation between work hours of teachers and student-teacher ratios [15]. In order to improve the working environment for school teachers, with the combined purpose of preventing the spread of COVID-19, the Japanese government has decided to reduce the upper-limit of class size, from 40 to 35, in primary schools after 2021.

The class-size reduction policy is expected to result in lowering student-teacher ratio (hereafter STR) because more teachers will be newly hired and assigned to schools. This study empirically addressed the research question of whether higher STR at a school level were associated with longer working hours and higher stress of school teachers. More specifically, this paper examined the association between STR, working hours and workload stress of school teachers, by using detailed teacher-level survey data obtained from TALIS 2018. Our empirical results suggested that the class-size reduction policy starting in 2021 could reduce teacher stress and long work hours through the consequent decrease in STR.

## Literature review

### Student-teacher ratio

Research on STR has a long history. An early representative example is The Tennessee Student/Teacher Achievement Ratio Study (STAR) Project and the group of subsequent studies using its survey data [16–19]. In addition, in the field of educational economics, there is a growing body of research on the effect of class-size reduction using quasi-experimental survey designs [20–23]. Most of these studies, however, aimed to examine the relationship between class size and student achievement, but not the relationship between class size and teacher stress or working hours. In addition, there is no theoretical analysis on the mechanism by which a decrease in STR reduces the working hours of teachers.

### Working hours and stress among teachers

As mentioned above, the long working hours of school teachers have become a serious issue in Japan [1, 8], but previous studies are limited. A Previous study showed a significant correlation between long working hours and psychological distress among male teachers, although the causal relationship was unclear [12]. The Ministry of Education, Culture, Sports, Science and Technology (MEXT) reported a positive and significant correlation between teachers’ working hours and STR [15].

The history of research on teacher stress is long, and studies have been reported in many countries [2, 25, 26]. In Japan, significant correlations were found between workload and depressive symptoms [10], workload stress and mental health [11], and both quantitative and qualitative workload and chronic fatigue [14]. These studies indicated that Japanese school teachers experienced high levels of stress in the workplace, which was detrimental to their physical and mental health, but their results seemed difficult to generalize because they were based on a survey of teachers working in schools in geographically limited region or city.

*Karoshi* is a Japanese term and can be translated as “overwork death”. Due to a national effort to reduce working hours, the number of Karoshi has been gradually decreasing from 317 in 2002 to 216 in 2019. However, there has been little improvement in the reduction of working hours for school teachers [27, 28]. Given the reduction of working hours and the following reduction in Karoshi in other professions, it is now vital to improve working hours and conditions for teachers in Japan.

## Methods

### Study sample

Data on working environment for teachers and STR in Japanese schools were obtained from TALIS 2018 dataset [29]. TALIS is an international, large-scale survey that asks teachers and school leaders about working conditions and learning environments at their schools. In order to obtain nationally representative sample of teachers for each ISCED (International Standard Classification of Education) level in each participating country and economy, a stratified two-stage probability sampling design was used [1]. As a result of this sampling, in Japan, 197 primary schools and 196 lower-secondary schools were sampled, and teachers working in the sampled schools participated in the survey. The number of teachers participated in the survey was 3308 for primary schools and 3555 for lower secondary schools. By excluding the teachers who were missing key variables used in the statistical analyses (see below), the study sample included 2761 and 3006 teachers for primary and lower-secondary school, respectively.

### Student-teacher ratios

STR was measured at the school level and obtained from the TALIS dataset file (variable named as “stratio” in the dataset). The ratio was derived by dividing the total number of students enrolled by the number of employed teachers in a given school [24].

### Work hours

Total working hours of teachers were obtained from the answers to the following question in teacher questionnaire: “During your most recent complete calendar week, approximately how many 60-minute hours did you spend in total on tasks related to your job at this school?”

Hours spent on individual tasks were obtained from the answers to the following question: “Approximately how many 60-minute hours did you spend on the following tasks during your most recent complete calendar week, in your job at this school?” Tasks are categorized into the following 10 types: a) Individual planning or preparation of lessons either at school or out of school; b) Team work and dialogue with colleagues within this school; c) Marking/correcting of student work; d) Counselling students (including student supervision, mentoring, virtual counselling, career guidance and behaviour guidance); e) Participation in school management; f) General administrative work (including communication, paperwork and other clerical duties); g) Professional development activities; h) Communication and co-operation with parents or guardians; i) Engaging in extracurricular activities (e.g. sports and cultural activities after school); j) Other work tasks [30].

### Workload stress and related variables

For the variables of workload stress, workplace well-being and stress, and job satisfaction, we utilized the relevant scale scores that were pre-derived and stored in the dataset (variable named as “t3wload”, “t3wels”, and “t3jsenv” in the dataset, respectively). According to the TALIS 2018 Technical Report [24], these variables were derived using latent modelling within the framework of confirmatory factor analysis based on the responses to the questions presented in Table 1. Note that for the variable of workplace well-being and stress, larger values indicate poorer workplace well-being and higher workplace stress.

**Table 1:** Item wording for workload stress, workplace well-being and stress, and job satisfaction scales

Workload stress (t3wload)	
Thinking about your job at this school, to what extent are the following sources of stress in your work?	
Response options: “Not at all” (1), “To some extent” (2), “Quite a bit” (3), “A lot” (4).	
A.	Having too much lesson preparation
B.	Having too many lessons to teach
C.	Having too much marking
D.	Having too much administrative work to do (e.g. filling out forms)
E.	Having extra duties due to absent teachers
Workplace well-being and stress (t3wels)	
In your experience as a teacher at this school, to what extent do the following occur?	
Response options: “Not at all” (1), “To some extent” (2), “Quite a bit” (3), “A lot” (4).	
A.	I experience stress in my work
B.	My job leaves me time for my personal life
C.	My job negatively impacts my mental health
D.	My job negatively impacts my physical health
Job satisfaction (t3jsenv)	
How strongly do you agree or disagree with the following statements?	
Response options: “Strongly disagree” (1), “Disagree” (2), “Agree” (3), “Strongly agree” (4).	
A.	I would like to change to another school if that were possible
B.	I enjoy working at this school
C.	I would recommend this school as a good place to work
D.	All in all, I am satisfied with my job

Source: [24]

### Statistical analysis

Our data about teachers were hierarchically nested within schools. Therefore, the appropriate analysis method for these data is multilevel-analysis. Because our interest in this study was in the average effects of STR and not in the heterogeneity of its effects across schools, we adopted a random intercept model as our method of analysis [31]. For the sampling weights, only level two weights (final school weights) were used [32]. Stata version 16.1 was used for estimation. The dependent variables were work hours, workload stress, workplace well-being, and job satisfaction. The key explanatory variable was STR. The other control variables were dummy variables for gender (Male: 0, Female: 1), employment status (Fixed-term: 0, Permanent: 1), years of teaching experience, and a dummy variable for school type (Public: 0, Private: 1).

## Results

### Sample characteristics

Descriptive statistics of the study sample were reported in Table 2. The mean of STR was 17.4 and 13.6 for primary school and lower-secondary school, respectively. Over 90 percent of teachers were permanently employed. The average work hours per week were 54.2 and 56.0 for primary school and lower-secondary school, respectively, and 30–40% of working hours were devoted to teaching. Among individual tasks, average hours spent on engaging extracurricular activities were 7.6 per week for lower-secondary school teachers, while those for primary school teachers were only 0.6. This difference reflected the fact that after-school club activities were widespread and enthusiastic in lower-secondary schools, and over 80% of lower-secondary school teachers engaged in the club activities as advisors [15].

**Table 2:** Descriptive statistics

	Primary school		Lower-secondary school	
	(*N*=2761)		(*N*=3006)	
	Mean	Std. Dev.	Mean	Std. Dev.
Student-Teacher ratio (STR)	17.393	5.041	13.596	4.338
Gender (Male: 0, Female: 1)	0.401	0.490	0.589	0.492
Employment status (Fixed-term: 0, Permanent: 1)	0.937	0.242	0.907	0.291
Teaching experience (Years)	16.759	11.845	17.037	11.805
School type (Public: 0, Private: 1)	0.015	0.121	0.101	0.301
Work hours per week (Hours)				
Total	54.152	14.237	55.970	18.132
Teaching	22.871	8.461	17.753	8.134
Preparation of lessons	8.507	7.184	8.369	7.001
Team work and dialogue with colleagues	4.068	3.355	3.606	3.430
Marking/correcting of student work	4.811	4.236	4.303	4.156
Counselling students	1.266	2.286	2.388	3.553
Participation in school management	3.140	5.432	2.868	5.012
General administrative work	5.122	6.345	5.568	6.757
Professional development activities	0.691	2.503	0.681	2.126
Communication and co-operation with parents	1.225	1.538	1.230	2.092
Engaging in extracurricular activities	0.570	1.970	7.586	7.686
Other work tasks	1.864	4.175	2.782	5.530
Workload stress	9.797	2.230	9.166	2.005
Workplace well-being and stress	9.225	2.128	9.334	2.155
Job satisfaction	12.297	1.922	11.998	1.954

### STR and work hours of teachers

The estimation results of the random intercept model with working hours as the dependent variable were presented in Table 3, but the results for explanatory variables other than STR had been omitted to save space. Fixed effect estimates of the slope of STR revealed that STR was positively correlated with total work hours and teaching hours. Among other tasks, hours spent on marking/correcting of student work, counselling students, and communication with parents or guardians were found to be positively correlated with STR. Also, in lower secondary schools, hours spent on extracurricular activities were found to have positive correlation with STR. These results confirmed that higher STR at a school level were associated with longer working hours of teachers.

**Table 3:** Fixed effect estimates of STR on work hours

	Primary school (*N*=2761)		Lower-secondary school (*N*=3006)	
Dependent variables	Coef. of STR	95% CI	Coef. of STR	95% CI
Total work hours	0.239***	[0.108, 0.370]	0.563***	[0.380, 0.745]
Teaching	0.084*	[0.002, 0.166]	0.266***	[0.194, 0.337]
Preparation of lessons	0.021	[-0.048, 0.091]	-0.021	[-0.090, 0.048]
Team work and dialogue with colleagues	0.092***	[0.060, 0.124]	0.029	[-0.006, 0.063]
Marking/correcting of student work	0.121***	[0.084, 0.158]	0.085***	[0.039, 0.131]
Counselling students	0.039***	[0.024, 0.053]	0.039*	[0.003, 0.075]
Participation in school management	-0.065**	[-0.113, -0.016]	-0.038	[-0.096, 0.020]
General administrative work	-0.128***	[-0.194, -0.062]	0.011	[-0.056, 0.077]
Professional development activities	-0.002	[-0.022, 0.018]	0.010	[-0.006, 0.027]
Communication and co-operation with parents	0.020**	[0.006, 0.034]	0.037***	[0.020, 0.053]
Engaging in extracurricular activities	0.008	[-0.013, 0.028]	0.096*	[0.008, 0.184]
Other work tasks	0.003	[-0.027, 0.034]	-0.013	[-0.069, 0.043]

Fixed effect estimates of STR obtained from estimations of the random intercept model were reported.

*** *p*<0.001, ** *p*<0.01, * *p*<0.05

### Teacher stress and related variables

Estimation results on teacher stress and related variables were reported in Table 4. STR was positively correlated with workload stress, while it was also positively correlated with workplace well-being and stress. The correlation between STR and job satisfaction was negative but statistically significant only for lower-secondary school teachers. These results confirmed that higher STR at a school level were associated with higher workload and workplace stress of teachers. For the other control variables, it was found that female teachers tended to report lower workload stress and lower workplace well-being than male teachers. On the other hand, teaching experience had no correlation with workload stress and related variables.

**Table 4:** Estimation results on workload stress and related variables

	Primary school			Lower-secondary school		
	Workload stress	Workplace well-being and stress	Job satisfaction	Workload stress	Workplace well-being and stress	Job satisfaction
Level 1 (Teacher)						
Intercept	7.254***	7.349***	13.366***	7.266***	7.711***	13.167***
	[6.666, 7.841]	[6.731, 7.968]	[12.811, 13.922]	[6.783, 7.749]	[7.313, 8.108]	[12.765, 13.569]
Gender (M: 0 F: 1)	-0.339***	-0.219*	0.141	-0.264**	-0.184*	0.104
	[-0.528, -0.150]	[-0.415, -0.022]	[-0.030, 0.313]	[-0.423, -0.104]	[-0.353, -0.016]	[-0.057, 0.265]
Employment status	1.842***	1.278***	-0.773***	1.147***	1.411***	-0.757***
(Fixed-term: 0 Permanent: 1)	[1.384, 2.300]	[0.895, 1.661]	[-1.090, -0.456]	[0.871, 1.424]	[1.141, 1.681]	[-1.032, -0.482]
Teaching experience (Years)	-0.002	0.006	-0.006	-0.001	-0.003	-0.003
	[-0.009, 0.005]	[-0.001, 0.014]	[-0.013, 0.002]	[-0.007, 0.005]	[-0.011, 0.006]	[-0.011, 0.005]
Level 2 (School)						
STR	0.058***	0.037**	-0.019	0.078***	0.040***	-0.037**
	[0.036, 0.080]	[0.013, 0.061]	[-0.040, 0.003]	[0.052, 0.104]	[0.019, 0.060]	[-0.060, -0.013]
School type	-0.313	0.545	-0.700	-0.222	-0.173	-0.023
(Public: 0 Private: 1)	[-0.684, 0.058]	[-0.277, 1.367]	[-1.968, 0.568]	[-0.623, 0.180]	[-0.483, 0.136]	[-0.318, 0.272]
Variance components						
Intercept variance	0.508***	0.482***	0.693***	0.415***	0.427***	0.550***
	[0.423, 0.611]	[0.392, 0.593]	[0.558, 0.861]	[0.333, 0.517]	[0.346, 0.527]	[0.440, 0.687]
Within-school variance	4.136***	3.897***	2.873***	3.343***	4.053***	3.220***
	[3.898, 4.389]	[3.636, 4.178]	[2.673, 3.088]	[3.119, 3.582]	[3.826, 4.293]	[3.006, 3.449]
Number of teachers	2761	2761	2761	3006	3006	3006
Number of schools	193	193	193	190	190	190

Estimation results of the random intercept model were reported. Figures in square brackets were 95% confidence intervals.

*** *p*<0.001, ** *p*<0.01, * *p*<0.05

We further tested interaction effects between STR and Gender and STR and Employment status and the three workload variables. Although estimation results were not reported, they revealed that these interaction effects were not statistically significant for most of the estimating equations. These results suggested that the effects of teacher gender and employment status on workload variables were independent of STR.

## Discussion

Our simple statistical analysis clearly showed that a higher STR at the school level was correlated with long working hours of teachers. The coefficient estimates revealed that, on average, lowering STR by five in lower-secondary school was associated with 2.8 hours shorter total working hours per week. In addition, it was found that teachers working at high-STR schools tended to spend much time not only on teaching but on the other tasks such as marking/correcting of student work, counselling students, and communication with parents or guardians. These results confirmed the results of previous studies [15]. Although these results did not indicate a strict causal relationship, our empirical results suggested that the working hours of teachers in Japan, the longest in the world, could be reduced by lowering STR at the school level.

Our statistical analysis also showed that teachers in high-STR schools tended to experience higher workload and workplace stress. In light of the fact that the number of teachers taking leave due to mental illness has remained high, lowering STR could have a positive impact on decreasing mental illness of teachers through reducing work hours and workload stress. The results of this study could be useful for those involved in dealing with issues related to teacher stress.

Since the sample analyzed in this study is considered to be a representative sample of teachers in Japan, the results of this study are considered to be widely valid within the country. In addition, since the problems of stress and burnout among school teachers have been observed not only in Japan but also in other countries, the empirical results on teacher stress and related variables may be generalizable, but further research using data from a wide range of countries will be necessary. On the other hand, as mentioned above, the working hours of school teachers in Japan are among the longest in the world, so it seems difficult to generalize the results on the relationship between STR and teachers’ working hours.

The limitation of this study was that it only showed the correlation between STR, working hours and teacher stress, and thus it did not identify a causal relationship between them. This was because the data used in this study did not employ an experimental design. If the local educational board, which had the discretion of assigning teachers to public schools, placed more teachers in schools where teachers had worked longer hours or where teachers had been more stressed, then the coefficient estimates reported above might be biased. However, even if such assignments of teachers had been made, the absolute value of the coefficient estimate of STR should be larger. In other words, the magnitude of the coefficient estimates of STR obtained in the above analysis would not be overestimated.

## Conclusion

In summary, this study underscores the association of a high student-teacher ratio with long working hours and high workload stress among school teachers in Japan. The class-size reduction policy starting in 2021 could reduce teacher stress and long work hours through the consequent decrease in student-teacher ratios.

## Data Availability

Data is publicly available and can be downloaded from the OECD website. http://www.oecd.org/education/talis/talis-2018-data.htm.

## Abbreviations

STR: Student-teacher ratios
OECD: Organisation for economic co-operation and development
TALIS: Teaching and learning international survey
MEXT: Japan’s ministry of education, culture, sports, science, and technology
